# Mitochondrial Quality Control and Metabolic Reprogramming in Hepatocellular Carcinoma: Implications for Immunotherapy and Treatment Resistance

**DOI:** 10.3390/cells15060517

**Published:** 2026-03-13

**Authors:** Yusra Zarlashat, Anna Picca

**Affiliations:** 1 Department of Biochemistry, Government College University Faisalabad, Faisalabad 38000, Pakistan; yusrazarlashat@gcuf.edu.pk; 2 Fondazione Policlinico Universitario “A. Gemelli” IRCCS, 00168 Rome, Italy; 3 Department of Medicine and Surgery, LUM University, 70010 Casamassima, Italy

**Keywords:** immune evasion, immunotherapy, metabolic reprogramming, mitochondrial biogenesis, mitochondrial dynamics, mitophagy, tumor microenvironment, T-cell exhaustion

## Abstract

Hepatocellular carcinoma (HCC) is a leading cause of cancer death, characterized by poor prognosis in advanced stages despite available therapies. Dysfunctional mitochondrial can initiate both tumor progression and antitumor immunity. Altered mitochondrial quality control mechanisms, including dynamics, biogenesis, and degradation, contribute to mitochondrial decline supporting hepatocarcinogenesis and tumor survival. Within the immunosuppressive tumor microenvironment, HCC cells shift their metabolism toward glycolysis, which reduces nutrient availability and triggers mitochondrial dysfunction in infiltrating immune cells, leading to T-cell exhaustion and weakened cytotoxic activity. Herein, we discuss how immune checkpoint inhibitors may respond to this exhaustion. While most findings showing that these therapies partially restore mitochondrial bioenergetics in T cells have been conducted in preclinical studies, direct clinical evidence in HCC patients remains limited. By combining current knowledge on mitochondrial metabolism, immune escape, and treatment resistance, we discuss how targeting mitochondrial pathways may help improve immunotherapy responses and support new combination treatment approaches against HCC.

## 1. Introduction

Hepatocellular carcinoma (HCC) is the most common type of primary liver cancer and ranks as the third leading cause of cancer-related death [1]. Despite existing treatments (e.g., liver transplantation, surgical resection, and molecular targeted therapies), its prognosis remains poor, especially in advanced stages. Five-year survival rates range from 25.9% to 41.7% in early-stage, 5.9% in intermediate-stage, and 0.2% to 0.4% in advanced-stage HCC. This is partly due to the ability of HCC cells to sustain proliferation, invasion, metastasis, and resistance to therapy. Hepatitis B/C virus (HBV/HCV) infection, metabolic dysfunction, and exposure to aflatoxin are major determinants for HCC development [2]. Chronic aflatoxin exposure has been reported to induce mutations in the TP53 gene, impairing tumor suppressor function and enhancing HCC risk, particularly in combination with HBV infection [3]. Beyond these traditional risk factors, environmental contaminants such as micro- and nanoplastics have recently been shown to favor mitochondrial dysfunction, oxidative stress, and lipid accumulation in human liver cells [4]. Additionally, epigenetic alterations, including DNA methylation, histone modifications, and noncoding RNA dysregulation, contribute to HCC pathogenesis by silencing tumor suppressor genes and are influenced by environmental exposures [5]. These factors collectively worsen mitochondrial dysregulation and genomic instability, further promoting HCC initiation and progression.

Evidence from mitochondrial biology indicate this organelle as a key contributor to hepatocarcinogenesis, with dysfunctional mitochondria supporting HCC progression by reprogramming cellular metabolism [6], generating oxidative stress to disrupt redox balance [7], and evading programmed cell death pathways [8]. Mutations in mitochondrial DNA (mtDNA) are observed in approximately 50% of HCC cases and can disrupt electron transport chain function and create metabolic vulnerabilities that can be prime targets for biogenesis agonists [9]. This intricate regulatory network highlights how disruption of mitochondrial health is a critical aspect of tumor initiation, progression, and ultimately patient survival. To maintain mitochondrial homeostasis and function, cells rely on a sophisticated mitochondrial quality control (MQC) system to monitor, repair, and/or selectively eliminate damaged mitochondria [10]. Dysregulation of these processes has also been implicated in liver pathology, influencing cell fate decisions, metabolic flexibility, and therapeutic resistance in HCC [11].

An immunosuppressive tumor microenvironment (TME) is a feature of HCC and represents a major barrier to effective treatment [12]. HCC cells undergo a metabolic switch, preferentially utilizing glycolysis over oxidative phosphorylation (OXPHOS) even when oxygen is available, a phenomenon called the Warburg effect. This metabolic reprogramming enables tumor cells to thrive in nutrient-depleted and hypoxic conditions but simultaneously starves infiltrating immune cells of critical resources, directly impairing their effector functions, promoting exhaustion, and inhibiting memory formation [13]. Several mechanisms may be in place linking tumor glycolysis to T-cell exhaustion. First, rapid glucose consumption by cancer cells creates a nutrient-depleted microenvironment, decreasing T cells from the glucose they need for glycolysis and effector function. Second, lactate accumulation from glycolytic tumors acidifies the TME, suppressing T-cell proliferation and cytokine production [14]. Third, the depletion of key metabolites, such as glutamine, further impairs T-cell activation and memory differentiation. Together, these metabolic limitations can prime T cells toward an exhausted phenotype characterized by sustained inhibitory receptor expression and reduced effector capacity [15].

Mitochondria are deeply involved in this immunometabolic cross-talk, initiating the bioenergetic and biosynthetic programs of both malignant and immune cells within the TME [16]. Furthermore, mutations in mitochondrial DNA (mtDNA), which can arise from oxidative damage caused by impaired bioenergetics, may disrupt the electron transport chain and further modify cellular metabolism. These changes can promote a pro-tumor environment that supports HCC development and metastasis [17].

Given this organelle’s central role, targeting mitochondrial pathways represents a promising, although underexplored, therapeutic strategy for HCC. In this view, MQC may be envisioned as a nexus for novel immune checkpoint inhibitor (ICI) synergies. Herein, we summarize current knowledge on how mitochondrial function, metabolic reprogramming, and antitumor immunity interact in HCC. We first describe how key MQC pathways, including dynamics, biogenesis, and degradation, are altered in HCC and how these changes contribute to tumor development. Next, we discuss how mitochondrial metabolism affects the function of immune cells in the TME, such as T cells, natural killer (NK) cells, and macrophages, as well as immunosuppressive cells like myeloid-derived suppressor cells (MDSCs) and regulatory T cells (Tregs). Lastly, we review therapies that target mitochondrial pathways, including ICIs, and highlight potential combination strategies to restore mitochondria in immune cells and improve patient outcomes.

## 2. Mitochondrial Quality Control Pathways in HCC

MQC systems are a set of interrelated housekeeping mechanisms, including biogenesis, degradation, and dynamics (fusion and fission), triggered in response to oxidative stress, nutrient deficiency, virus infection, abnormal metabolite accumulation, hypoxia, and other pathophysiological factors. This ensures that enough functional mitochondria meet cellular needs in the context of cell damage [10].

### 2.1. Mitochondrial Dynamics

**Figure 1:**
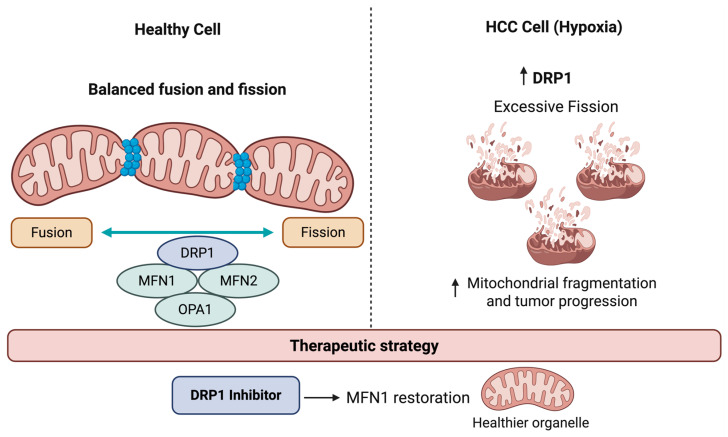
Mitochondrial dynamics in normal hepatocytes and HCC cells. Normal cells maintain balanced mitochondrial fusion and fission processes, supporting efficient energy production. In contrast, cancer cells show increased mitochondrial fission initiated by DRP1, leading to enhanced mitochondrial fragmentation and tumor progression. Abbreviations: DRP1, dynamin-related protein 1; MFN, mitofusin; OPA1, optic atrophy 1. Created with Biorender.com.

Mitochondrial fusion and fission are essential processes preserving organelle’s health. Fusion allows dysfunctional mitochondria to merge with healthy counterparts, complementing and buffering damage while ensuring mitochondrial DNA integrity. Fission divides mitochondria into smaller fragments, tagging damaged portions for elimination [18]. Dynamin-related protein 1 (DRP1) is the main mediator of fission, whereas two dynamin-like GTPases, mitofusin 1 and 2 (MFN1/MFN2), along with optic atrophy 1 (OPA1) and cardiolipin, enable fusion of the inner and outer mitochondrial membranes [19]. Together, these opposing processes maintain a healthy mitochondrial network by generating new organelles and facilitating the removal of damaged ones [10] (Figure 1).

Both abnormal mitochondrial fusion and fission have been reported in HCC [20]. Zhang et al. [21] showed that HCC progression is driven by a distinct pro-fission mitochondrial state, orchestrated by upregulation of mitochondrial fission regulator 2 (MTFR2) and DRP1. This state promotes tumor aggression, stemness, TP53 mutations, and altered immune responses, confirming that dysregulated mitochondrial dynamics, and specifically excessive fission, are central to HCC pathogenesis (Figure 1). Targeting the MTFR2-dependent fission pathway may therefore offer a therapeutic option to restore mitochondrial homeostasis and improve outcomes in HCC. However, evidence is primarily from experimental models, including in vitro studies and mouse xenografts.

In another study, Sun et al. [22] showed that HCC cell migration and metastasis are initiated by a distinct pro-fission state, characterized by the upregulation of DRP1 and downregulation of MFN1. This state promotes tumor aggression by reprogramming focal-adhesion dynamics and lamellipodia formation, primarily through activation of the Ca^2+^/CaMKII/ERK/FAK (calcium ion/calcium-calmodulin-dependent protein kinase II/extracellular signal-regulated kinase/focal adhesion kinase) pathway. These findings confirm that dysregulated mitochondrial dynamics, specifically excessive fission, are central to HCC pathogenesis and progression. Key cellular processes, such as proliferation, secretion, differentiation, and apoptosis, which initiate tumor growth and survival, are also regulated through metabolic reprogramming in HCC [23]. However, the precise link between these pathways, mitochondrial function, and cellular homeostasis in HCC remains unclear. Key unresolved questions include how mitochondrial dynamics (fission/fusion) are coordinated with metabolic signaling pathways, whether mitochondrial defects are a cause or consequence of HCC progression, and how MQC mechanisms differentially impact tumor cells versus immune cells within the TME. Beyond energy production, mitochondria in HCC cells influence iron metabolism and control cell cycle progression, thereby contributing to tumor development [24]. Additionally, mitochondria are crucial in regulating inflammation and triggering inflammatory disorders by affecting innate immune responses [25].

### 2.2. Mitochondrial Biogenesis

Nuclear transcription factors such as nuclear respiratory factor 1 and 2 (NRF1/NRF2) and nuclear co-activators (i.e., peroxisome proliferator-activated receptor gamma coactivator 1α (PGC-1α)) regulate mitochondrial biogenesis [26]. Mitochondrial biogenesis markers are significantly altered in HCC compared to normal liver tissue. Analysis from clinical samples and publicly available datasets, including The Cancer Genome Atlas (TCGA), reveal consistent downregulation of key mediators (Table 1).

Data compiled from TCGA database and published studies indicate that mitochondrial transcription factor A (TFAM) expression shows variability across HCC samples, with some studies reporting similar levels between tumor and normal tissues in 18 of 20 paired samples, while only 2 HCC tissues showed a 1.8-fold increase [30]. However, functional impairment of TFAM in HCC is supported by evidence that TFAM depletion induces AMP-activated protein kinase (AMPK) activation, reduces mitochondrial respiration, and increases sensitivity to chemotherapeutic agents.

NRF1 and NRF2 regulate this process by encoding TFAM and electron transport chain subunits. TFAM binds to mtDNA and regulates both mtDNA replication and transcription initiation, as well as mtDNA maintenance [31]. PGC-1α, though it does not directly bind to DNA, is recruited to chromatin and functions as a regulator by activating transcription factors and/or interacting with nuclear receptors to promote mitochondrial biogenesis [26]. In HCC, decreased mitochondrial biogenesis and oxidative phosphorylation have been associated with tumor progression and the NRF1/PGC1α axis has been indicated as a potential promising target for improving the sensitivity of HCC treatment [32].

Pharmacological activation of mitochondrial biogenesis represents an emerging therapeutic strategy. Exercise mimetics such as AICAR (5-aminoimidazole-4-carboxamide ribonucleotide), an AMPK agonist, have been shown to enhance PGC-1α expression and promote mitochondrial biogenesis in various models [33]. In preclinical studies, AICAR treatment increases mtDNA content, upregulates OXPHOS complex expression, and improves mitochondrial respiratory function [34]. Importantly, AICAR has been shown to be able to restore OXPHOS activity by approximately 25% in models of mitochondrial dysfunction, suggesting therapeutic potential for metabolic diseases and cancer. AICAR also exerts anti-cancer effects by switching cancer cells from glycolytic metabolism toward oxidative phosphorylation, thereby reducing lactate production and increasing sensitivity to chemotherapy agents such as doxorubicin [35].

However, it is important to note that enhanced mitochondrial biogenesis also supports tumor survival in certain contexts. Some studies have shown that increased PGC-1α activity in cancer cells promotes metabolic flexibility, allowing them to adapt to nutrient-poor conditions and resist therapy-induced stress. Therefore, targeting the NRF1/PGC-1α axis may have context-dependent effects, and the timing and tumor stage should be considered when developing such therapeutic strategies.

### 2.3. Mitochondrial Degradation

Several mechanisms work synchronously to break down and/or eliminate mitochondria. The ubiquitin–proteasome system mediates the degradation of some outer mitochondrial membrane proteins, whereas AAA ATPase family proteases, which are found on both sides of the inner mitochondrial membrane (IMM), can break down proteins in the matrix and intermembrane space and are responsible for the degradation of IMM proteins [36]. More recently, a unique process operating through the production of vesicles by the IMM that selectively removes damaged mitochondrial portions of IMM has been identified and likely preserves the structure and composition of normal mitochondrial cristae, possibly shielding mitochondria from localized damage [37]. Part of mitochondria breakdown also occurs via mitochondria-derived vesicles (MDVs), carrying oxidized proteins to peroxisomes and/or lysosomes for final clearing [38]. Completely damaged mitochondria, instead, are packed into migrasomes or microvesicles and released from cells as extracellular vesicles via mechanisms including mitocytosis and autophagic secretion of mitochondria [39]. Additionally, either direct lysosome-mediated micromitophagy or mitophagy, which requires autophagosome formation, can be used to selectively remove damaged mitochondria. The PINK1–Parkin pathway represents a conserved canonical mitophagy mechanism wherein mitochondrial damage triggers PINK1 accumulation on the outer mitochondrial membrane, which subsequently recruits and activates Parkin to ubiquitinate mitochondrial proteins, marking them for autophagosomal engulfment and lysosomal degradation [40].

MQC pathways are frequently dysregulated in HCC models, contributing to tumorigenesis [6]. Specifically, mitophagy is often upregulated in HCC cells as a pro-survival mechanism, enabling them to eliminate damaged mitochondria and evade apoptosis under hypoxic and metabolic stress conditions within the TME [41]. However, it is important to distinguish between the context-dependent dual roles of mitophagy in HCC. In early stages of tumor development, impaired PINK1–Parkin-mediated mitophagy may promote HCC progression by allowing damaged mitochondria to accumulate, leading to oxidative stress and inflammatory signaling that initiate malignant transformation [42]. Alterations in MDV formation have also been implicated in modulating redox balance and inflammatory responses in pre-malignant hepatocytes. In contrast, in established tumors, increased mitophagy may act as a pro-survival mechanism that allows cancer cells to adapt to metabolic stress by removing damaged mitochondria and preventing apoptosis.

This hyperactivated mitophagy in advanced HCC has been reported to contribute to sorafenib resistance by clearing drug-damaged mitochondria and promoting cancer cell survival. Importantly, inhibiting mitophagy flux with lysosomal inhibitors such as bafilomycin A1 or chloroquine has been shown to sensitize resistant HCC cells to sorafenib, enhancing antitumor efficacy in preclinical models [43]. Studies have shown that blocking autophagic degradation with bafilomycin A1 significantly increases sorafenib-induced cell death and apoptosis activation under hypoxia-mimicking conditions [44]. Similarly, inhibition of the PINK1–Parkin pathway via genetic knockdown or pharmacological inhibition enhances sorafenib and regorafenib sensitivity in HCC cells and in vivo tumor models, suggesting that targeting mitophagy represents a promising therapeutic strategy to overcome drug resistance [43].

Mitochondria can also change their shape under certain signals and undergo self-degradation by acquiring lysosomal components. This process involves the formation of specialized structures, including mitochondria-lysosome-related organelles (MLROs) and mitochondrial spheroids [40]. MLROs form when mitochondria-derived vesicles fuse with lysosomes and are regulated by transcription factor EB (TFEB). Unlike other mitochondrial vesicles, MLROs can degrade all mitochondrial components, including the outer and inner membranes and matrix proteins [45]. These alternative quality control pathways operate independent of canonical mitophagy and represent additional therapeutic targets in HCC [46].

Additionally, the activation of MLROs is related to the dedifferentiation of adipocytes and hepatocytes in mice models of HCC. The potential significance of MLROs in many liver diseases is highlighted by the fact that hepatocyte dedifferentiation is frequently seen in the last stages of chronic liver diseases, leading to liver failure. Importantly, hepatocyte dedifferentiation is a hallmark of hepatocarcinogenesis, suggesting that MLRO-related pathways activated in HCC contribute to a cellular context that promotes the initiation and progression of HCC [11]. Together, mitochondrial spheroids, vesicles derived from the IMM, and MLROs represent the outcome of distinct mitochondrial dynamics that arise in response to oxidative mitochondrial damage, independent of classical processes.

Several studies have established a connection between mitophagy and liver diseases, including metabolic dysfunction-associated steatotic liver disease (MASLD) and viral hepatitis, making mitophagy a primary pathway for mitochondrial breakdown in this setting. Hepatocytes in MASLD show mitochondrial depolarization and early mitochondrial dysfunction that ultimately triggers mitophagy [47]. Additionally, HBV/HCV-induced mitochondrial mitophagy and acute/chronic alcohol exposure reduce hepatic damage and inflammation in multiple models [48]. In HCC, mitophagy suppresses tumor initiation by eliminating damaged organelles; its sustained activation supports cancer cell survival, metabolic adaptation, and resistance to therapy [49]. Recent studies also revealed that specific inhibition of mitophagic flux sensitizes HCC cells to chemotherapeutic agents, positioning mitochondrial degradation pathways as promising therapeutic targets for HCC [50].

Altogether, these studies indicate that the pathophysiology of several liver diseases, including MASLD, viral hepatitis, and HCC, is influenced by deficient quality control systems and impaired mitochondrial activity, and MQC may represent a relevant target for restoring organelle homeostasis in response to mitochondrial damage and/or energy deprivation.

## 3. Mitochondrial Regulation in Immune Cells of the Tumor Microenvironment

Mitochondrial metabolic reprogramming defines an adaptive modification of mitochondrial metabolic pathways and functions, often triggered by changes in the cellular environment or physiological state [51]. Stress conditions in the TME, such as high production of reactive oxygen species (ROS), decreased oxygen consumption, depolarized membrane potential, and defective biogenesis, affect mitochondrial metabolism and immune cell’s function. Mitochondrial metabolic reprogramming plays a key role in regulating the anticancer activity of immune cells that invade the TME [52]. The metabolic profiles of key immune cells in the healthy state, their dysfunction within the HCC TME, and potential strategies to restore their antitumor activity are summarized in Table 2.

Among the immune cells, T cells recognize and eliminate cancerous cells. When T cells are activated, their metabolism changes from OXPHOS to glycolysis, which enables them to proliferate quickly and perform effector activities [59]. This glycolytic shift characterize effector T cells, which prioritize rapid proliferation and cytokine production over energy efficiency. In contrast, memory T cells rely primarily on OXPHOS and fatty acid oxidation (FAO) for long-term survival and rapid recall responses. When T cells infiltrate tumors, they exhibit a sustained decrease in mitochondrial mass and function. Additionally, B-lymphocyte-induced maturation protein 1-mediated inhibition of PGC-1α-dependent mitochondrial reprogramming results from ongoing T cell activation in hypoxic conditions [60]. However, studies reported that T cell function is improved by increasing mitochondrial metabolism. This is particularly relevant for generating memory T cells, as they rely on robust mitochondrial function. Overexpression of PGC-1α enhances the antitumor activity of CD8^+^ T cells by boosting their metabolic capacity and promoting mitochondrial biogenesis. Separately, activation of the T-cell surface receptor 4-1BB starts mitochondrial fusion and biogenesis in CD8^+^ tumor-infiltrating lymphocytes (TILs) through mechanisms that do not depend on PGC-1α or p38-MAPK signaling [61].

NK cells are important cytotoxic cells that contribute to the immune response against altered or infected cells [62]. When NK cells are dormant, they mostly produce ATP by mitochondrial OXPHOS; when they are activated, glycolysis and OXPHOS both rise, further increasing ATP production [63]. The potential of NK cells to inhibit the development of B16F10 melanoma in vivo was shown to be severely restricted by a reduction in PGC-1α, an important regulator of mitochondrial biogenesis, showing the critical role of mitochondria in NK cells [64]. NK cells have serious disadvantages after infiltrating the TME. NK cells derived from liver tumors show dysfunctional mitochondria and a reduced metabolism with low glycolysis. Quantitative analysis reveals that NK cells from HCC patients show approximately 60% reduction in glycolytic capacity compared to healthy controls, correlating with impaired cytotoxic function and poor clinical outcomes [65].

In patients with HCC, elevated transforming growth factor-β (TGF-β) in the TME contributes to NK cell dysfunction, with circulating NK cells exhibiting transcriptional and phenotypic signatures of metabolic impairment. In these cells, reduced glycolytic and oxidative metabolic capacity, along with hypoxia-related mitochondrial fragmentation, were identified and correlated with impaired effector function. Moreover, blockade of TGF-β partially restores aspects of NK cell metabolism and activity in experimental models [66]. Upon functional allogeneic mitochondrial transplantation, NK cells showed a notable increase in immunological activity and cytotoxicity, suggesting that improved mitochondrial function can restore NK cell activity within the TME. Combining mitochondrial-targeted strategies with immunostimulatory cytokines such as interleukin 15 (IL-15), which enhances both glycolysis and OXPHOS in NK cells, represents a promising approach to strengthen NK-mediated antitumor immunity in HCC [67]. Mitochondrial transplantation, though, while proving successful in experimental models of cancer, warrants investigation in HCC.

Mitochondria regulate immunological responses, signaling cascades, and metabolic reprogramming during macrophage development and function. M1 macrophages, one of the two macrophage phenotypes, release proinflammatory cytokines such as IL-1b, IL-6, and IL-12 to support antitumor immunity [68]. Like the Warburg effect of cancer cells, M1 macrophages preferentially use glycolysis to produce ATP in the presence of oxygen. This glycolytic metabolism in M1 macrophages is supported by fragmented mitochondria with reduced oxidative phosphorylation, a process regulated by MQC mechanisms including fission and mitophagy. The M2-like phenotype is seen in the majority of TME macrophages [69]. This is because macrophages undergo M2 polarization because of low glucose, high lactate, and hypoxic environment of the TME. Conversely, M2 macrophages rely on oxidative phosphorylation and require intact mitochondrial networks with efficient quality control to sustain their anti-inflammatory functions. Additionally, tumors release chemokines and cytokines, including TGF-β, IL-10, colony-stimulating factor 1 (CSF-1), and C-C motif chemokine ligand 2 (CCL2), which recruit macrophages and promote their polarization toward the M2 phenotype [70]. Thus, MQC pathways play a key role in determining macrophage polarization and function within the TME.

Finally, programmed cell death pathways including apoptosis, ferroptosis, and cuproptosis are also intimately linked to mitochondrial integrity and influence responses to immune checkpoint inhibitors. Apoptosis is triggered by mitochondrial outer membrane permeabilization and cytochrome c release, while ferroptosis involves mitochondrial lipid peroxidation and iron accumulation [71]. Dying tumor cells release damage-associated molecular patterns that activate NK cells, macrophages, and dendritic cells (DCs), enhancing antitumor immunity.

## 4. Mitochondrial Metabolism in Immunosuppressive Cells

**Figure 2:**
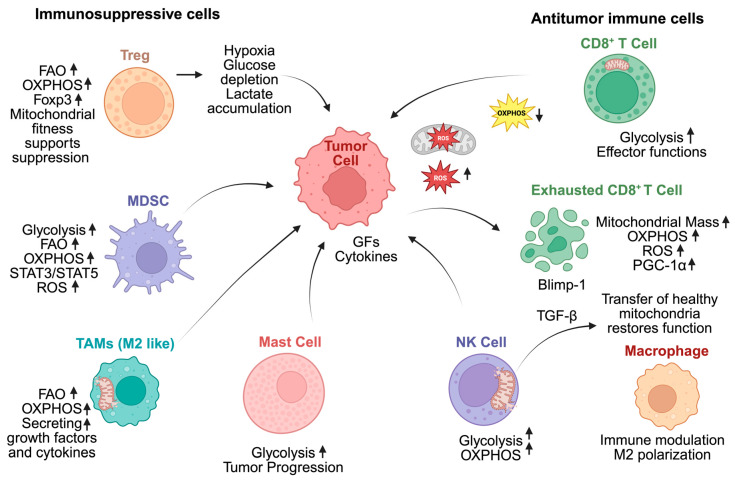
Changes in mitochondrial metabolism affects immune and immunosuppressive cells in the tumor microenvironment. Tumor cells create a stressful environment by consuming large amounts of glucose and producing lactate, which leads to hypoxia, nutrient shortage, and increased ROS. These conditions disrupt mitochondrial function in nearby immune cells, reducing their ability to combat HCC. Abbreviations: Blimp-1, B lymphocyte-induced maturation protein-1; CD8^+^ T cell, cluster of differentiation 8 positive T cell; FAO, fatty acid oxidation; Foxp3, forkhead box P3; GFs, growth factors; M2, alternatively activated (M2) macrophages; MDSC, myeloid-derived suppressor cell; NK cell, natural killer cell; OXPHOS, oxidative phosphorylation; PGC-1α, peroxisome proliferator-activated receptor gamma coactivator-1 alpha; ROS, reactive oxygen species; STAT, signal transducer and activator of transcription; TAMs, tumor-associated macrophages; TGF-β, transforming growth factor beta; Treg, regulatory T cells. Created with Biorender.com.

The TME is composed of many immunosuppressive cells that support immune evasion and carcinogenesis. These consist of mast cells, MDSCs, Tregs, and tumor-associated macrophages (TAMs) [72] (Figure 2). Several studies have shown the relevance of mitochondrial metabolism and functions in immunosuppressive cells, indicating also a role as targets for treatment [73].

Tregs are immunosuppressive cells that inhibit the activity of different immune cells, such as CD4^+^ T helper cells, CD8^+^ cytotoxic T cells, and NK cells. Tregs mainly depend on FAO and OXPHOS for their energy requirements. Forkhead box P3 (Foxp3) expression increases in the TME, where lactate is available, and glucose is limited. This inhibits Myc and glycolysis and supports OXPHOS and Tregs in adapting to their surroundings [74]. Hypoxia-inducible factor 1-alpha (HIF-1α) acts like a metabolic switch in TME, alternating between immunosuppression in Tregs caused by OXPHOS and migration controlled by glycolysis [75]. Tregs use fatty acid production to support their functional maturation after absorbing and storing fatty acids as lipid droplets. When fatty acid-binding protein 5 is inhibited in Tregs, mtDNA can be released and triggers type I interferon signaling via cyclic GMP–AMP synthase (cGAS)/stimulator of interferon genes (STING) pathway. This mechanism inhibits Treg activity and promotes the synthesis of the regulating cytokine IL-10 [76].

Pharmacologic inhibition of FABP5 in Tregs triggers mitochondrial changes including decreased OXPHOS, impaired lipid metabolism, and loss of cristae structure, leading to mtDNA release and consequent cGAS-STING-dependent type I interferon signaling that induces increased production of IL-10. This has been reported in tumor-infiltrating Tregs and may underlie enhanced immunosuppression in the TME [76]. Importantly, FABP5 inhibition reduces IL-10 production by approximately 70%, representing a potential strategy to limit Treg-mediated immunosuppression [77]. Combining FABP5 inhibitors with FAO blockers such as etomoxir, which targets the mitochondrial rate-limiting enzyme carnitine palmitoyl transferase 1A (CPT1A), the critical gatekeeper controlling fatty acid entry into mitochondrial oxidation and decreases Treg accumulation, represents an intervention strategy to disrupt the dual immunomodulatory functions of FAO within tumors [78]. However, recent evidence suggests that etomoxir’s effects on T cells may involve mechanisms beyond CPT1A inhibition, including direct effects on mitochondrial respiration, warranting careful interpretation of combination studies.

Neutrophil extracellular traps (NETs) promote Treg differentiation by enhancing mitochondrial respiration, thereby strengthening immunosuppression in MASH-HCC [79]. Mast cells release mtDNA and mitochondrial particles that trigger inflammation through autocrine and paracrine signaling [80]. Mitochondrial dynamics govern M1/M2 macrophage polarization: M1 cells use fragmented mitochondria and glycolysis, while M2 cells require intact networks for OXPHOS and anti-inflammatory functions [69].

MDSCs are neutrophils and immunosuppressive monocytes that mainly rely on glucose metabolism. The stimulation of β-adrenergic receptor promotes the signal transducer and activator of transcription 3 (STAT3) pathway, which improves MDSC survival by increasing OXPHOS and glutamine consumption by the tricarboxylic acid (TCA) cycle while reducing mitochondrial ROS via NRF2 signaling [81]. Smaller tumors, lower MDSCs, and enhanced antitumor immune response are the effects of decreased glucose availability and lactate generation [82]. MDSCs also depend on lipid metabolism. Tumor-derived cytokines increase MDSC lipid transport receptors and activate the STAT3 and STAT5 pathways [83]. This improves oxidative metabolism, lipid uptake, and immunosuppressive properties of MDSCs [84]. The immunosuppressive properties of MDSCs are reduced by blocking fatty acid intake and FAO [85]. Pharmacologic inhibition of FAO with etomoxir has been shown to block immune inhibitory pathways in tumor-infiltrating MDSCs, decrease their production of immunosuppressive cytokines, and significantly delay tumor growth in a T-cell-dependent manner [77]. FAO inhibition combined with low-dose chemotherapy blunted MDSC immunosuppressive effects and induced significant antitumor responses.

TAMs, which are mostly represented by a M2 phenotype, release growth factors, chemokines, and cytokines to develop tumor and metastasis [86]. TAMs reduce the pentose phosphate pathway and glycolysis in TME while shifting their metabolism toward OXPHOS and FAO. Immunosuppressive signaling is promoted by this metabolic reprogramming, which is advantageous for tumor development in the TME [87]. When stimulated, mast cells quickly degranulate and release their granule content into the extracellular space, inducing them to produce a set of chemokines and cytokines that cause inflammation through exocytosis [88]. The release of mtDNA and mitochondrial particles induce the production of cytokines and promote inflammation through autocrine or paracrine signaling [89]. Altogether, these findings indicate that immunosuppressive mechanisms and mitochondrial metabolic processes interact closely within the TME, suggesting possible targets for therapeutic approaches that boost antitumor immune responses.

## 5. Mitochondrial Metabolism of Cancer Cells and Immune Evasion

**Figure 3:**
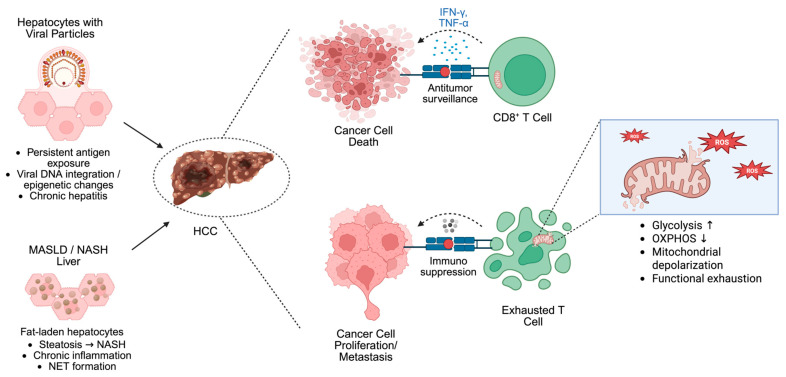
T cells initiate a powerful and expanding immune response upon recognizing a tumor or viral antigen, rapidly differentiating to carry out their defensive functions. However, chronic antigenic stimulation from a persistent HBV/HCV infection triggers a dysfunctional state known as exhaustion. This exhaustion weakens the antiviral response and dampens subsequent antitumor immunity, a process initiated by defective mitochondria within the immune cells. These damaged mitochondria fail to meet the sustained metabolic demands of an active immune cell, leading to T cell exhaustion characterized by an inability to self-renew and sustain continuous differentiation. Abbreviations: CD8^+^ T cell, cluster of differentiation 8 positive T cell; HBV, hepatitis B virus; HCC, hepatocellular carcinoma; HCV, hepatitis C virus; IFN-γ, interferon gamma; MASLD, metabolic dysfunction-associated steatotic liver disease; NASH, non-alcoholic steatohepatitis; NET, neutrophil extracellular trap; OXPHOS, oxidative phosphorylation; TNF-α, tumor necrosis factor alpha. Created with Biorender.com.

Metabolic alterations of cancer cells profoundly influence immunity in the HCC TME [90] (Figure 3). The Warburg effect, in which tumor cells greatly increase their intake of glucose and generation of lactate in the presence of oxygen, is one of the best-known processes of metabolic reprogramming [91]. Tumor-infiltrating immune cells have less access to glucose due to glycolytic activity and increased glucose absorption by cancer cells, which would encourage immunological escape [92]. Additionally, a significant relationship exists between enhanced programmed death-ligand 1 (PD-L1) expression and higher glycolytic activity in tumor cells [93]. Current evidence indicates that glycolysis initiates PD-L1 expression. The glycolytic metabolite lactate and the acidic TME have been shown to upregulate PD-L1 on tumor cells and immune cells through multiple mechanisms, including HIF-1α-dependent signaling [94]. Lactate is one of the main metabolites produced by glycolysis, helping cancer cells evade the immune system. Lactate suppresses DC maturation and impairs their antigen-presenting function by reducing the expression of major histocompatibility complex class II molecules [95]. Lactate diminishes the efficacy of cytotoxic T lymphocytes (CTLs) by suppressing the secretion of pro-inflammatory cytokines such as interferon-γ (IFN-γ), which are essential for CTL-mediated antitumor responses, and by interfering with the release of cytolytic effector molecules [96]. Tumor-derived lactate impairs NK cell function by directly suppressing their cytotoxic activity, downregulating activating receptors such as NKp46, and promoting NK cell apoptosis, while also indirectly expanding MDSCs that further inhibit NK-mediated cytotoxicity [97].

The power of HCC cells to evade the immune system is positively correlated with mitochondrial activity. In HCC cells, depletion of N-acetyltransferase 1 has been associated with reduced expression of OXPHOS and mitochondrial biogenesis-related proteins, accompanied by increased levels of antigen presentation-associated proteins [98]. Glucose transporter 1 (GLUT1) and monocarboxylate transporter 1 (MCT1) levels are linked to higher cluster of differentiation 147 (CD147) expression, which is increased in several malignant tumors. GLUT1 and MCT1 upregulation directly promotes PD-L1 expression through lactate-mediated HIF-1α stabilization, creating a feed-forward loop that may enhance immune evasion [99]. This metabolic-immunoregulatory axis can be therapeutically targeted by combining glucose uptake inhibitors with lactate dehydrogenase A inhibitors such as FX11, which have been shown to reduce lactate production, decrease PD-L1 expression, and restore T-cell cytotoxicity in preclinical HCC models.

This increased glycolytic metabolism in HCC cells has also been associated with the infiltration of immunosuppressive lymphocytes in HCC tissues [100]. Additionally, cancer cells interfere with the mitochondrial metabolism of immune cells. For instance, HCC secretes alpha-fetoprotein, which lowers PGC1-α and sterol regulatory element-binding protein 1 (SREBP-1) in DCs, resulting in decreased ATP synthesis, oxygen consumption rate, and lipogenesis [101].

### Mitochondrial Transfer to the Tumor Microenvironment

A recently discovered mechanism of immune evasion involves the direct transfer of mitochondria from immune cells to cancer cells within the TME [102]. This phenomenon has been primarily observed in experimental systems, including co-culture models and mouse studies, where mitochondrial transfer from T cells enhances cancer cell cycle progression and upregulates genes involved in energy production, cytoskeletal remodeling, and tumor necrosis factor-α (TNF-α) signaling pathways. This transfer can occur via multiple mechanisms, including tunneling nanotubes (TNTs), extracellular vesicles (EVs), and bacteriophages.

Immune cells, particularly T cells and macrophages, can extend direct TNTs to HCC cells, allowing bidirectional transfer of functional mitochondria enabling immune evasion and metabolic reprogramming [103]. HCC cells can also acquire mitochondria through EV-mediated transfer, with stressed or damaged mitochondria being packaged into EVs and delivered to neighboring cancer cells [104].

Functionally, mitochondria acquired from immune cells integrate into the existing mitochondrial network of HCC cells, boosting oxidative phosphorylation capacity, ATP production, and proliferative potential. This transfer also promotes metabolic flexibility, allowing cancer cells to survive under nutrient-deprived conditions and resist therapy-induced stress [54]. While these findings are compelling, direct evidence of mitochondrial transfer from immune cells to cancer cells in patient-derived HCC samples remains limited and requires further investigation to confirm its occurrence in human tumors. Alongside these effects, cancer cells further reprogram their metabolism, most notably through the Warburg effect, to support proliferation and resist immune-mediated clearance.

Bacteriophages (phages) have also been engineered to deliver therapeutic agents specifically to the TME, offering targeted delivery with reduced systemic toxicity. Phage-based delivery systems can be modified to display tumor-specific ligands, enabling precise targeting of HCC cells while sparing healthy tissues [105].

Exosomes derived from various cell types provide also a biocompatible platform for delivering therapeutic cargo, including mitochondria-targeted drugs, microRNAs (miRNAs), and proteins, to HCC cells [106]. These natural nanovesicles exhibit low immunogenicity, high stability in circulation, and the ability to cross biological barriers, making them attractive vehicles for targeted therapy [107]. Engineering exosomes to display HCC-specific ligands on their surface further enhances tumor accumulation and cellular uptake [106].

miRNAs and long non-coding RNAs have also emerged as key regulators of HCC pathogenesis and therapeutic targets. miRNA-based strategies involve either restoring tumor-suppressive miRNAs (such as miR-34a, miR-122, and miR-125a-5p) or inhibiting oncogenic miRNAs (such as miR-21, miR-221, and miR-660-5p) using antisense oligonucleotides or miRNA sponges [108]. Long non-coding RNAs, including homeobox transcript antisense RNA, metastasis-associated lung adenocarcinoma transcript 1, and highly upregulated in liver cancer, modulate gene expression by interacting with chromatin-modifying complexes, sponging miRNAs, or regulating mRNA stability, and their dysregulation contributes to HCC proliferation, metastasis, and chemoresistance [109].

These delivery systems exploit the unique properties of cancer cell mitochondria such as their elevated membrane potential and increased reactive oxygen species to enhance drug accumulation and therapeutic efficacy. Combining phage-based targeting, exosome-mediated delivery, and RNA-based therapeutics represents a promising multi-pronged approach for HCC treatment [110].

## 6. Targeting T-Cell Mitochondria in HCC Progression

Although several therapeutic approaches have been implemented for HCC, including radiofrequency ablation, liver transplantation, chemotherapy, surgical resection, and molecular targeting, the efficacy of these treatments on HCC progression is still inadequate [111]. In immunotherapy, immune cells identify and target cancer cells, and immunometabolism, which is emerging as a key therapeutic target through the regulation of mitochondria, the central hubs of cellular metabolic pathways (Figure 3).

### 6.1. Viral Hepatitis-Induced HCC

Active HBV/HCV infection is increasingly recognized as a primary cause of HCC. Specifically, HBV induces MASH-like HCC without cirrhosis. Long-term interactions between the hepatitis virus and hepatocytes induce epigenetic dysregulation of tumor suppressor genes and/or DNA integration, contributing to the conversion of HBV/HCV infection to HCC [107]. The immune response of the host to persistent chronic hepatitis B infection unfolds through five phases: immunological tolerance, inactive disease, active disease, immune-reactive phase, and progression to HCC. Throughout these phases, it is important to distinguish between two distinct roles of CD8^+^ T cells: their antiviral function aimed at clearing virus-infected hepatocytes and their antitumor function aimed at recognizing and eliminating malignant cells. While both responses involve CD8^+^ T cells and can become exhausted over time, they target different antigens and are influenced by different factors within the liver microenvironment. Viral persistence initiates antiviral T-cell exhaustion, whereas tumor development requires antitumor T cells to overcome additional immunosuppressive barriers specific to the tumor [86]. CD8^+^ T cells perform both functions of adaptive immunity in HBV-related HCC patients at every stage of hepatocarcinogenesis [112].

Furthermore, CD8^+^ T cells are essential for the removal of HBV, possibly by direct cytotoxicity via IFN-γ-mediated non-cytopathic lesions. In contrast, during persistent chronic HBV infection (CHB), T cells undergo continuous antigenic stimulation. As a result, antiviral CD8^+^ T cells are lacking, fail to develop into functional memory T cells, and HBV-specific T cells progressively become exhausted [113]. Long-term HBV infection gradually reduces the effector HBV-specific CD8^+^ T cells, which eventually weakens the adaptive immune system’s tumor surveillance, which encourages cancer cells to evade the immune system and accelerates the development of carcinogenesis [112]. In HCV infection, T-cell exhaustion is associated with elevated expression of glycolysis-related genes and weakened mitochondrial function in HCV-specific CD8^+^ T cells [114]. Schurich et al. [115] showed that HBV-specific T cells exhibit mitochondrial dysfunction, characterized by non-functional large mitochondria and reduced mitochondrial potential, which restricts the metabolic remodeling of exhausted T cells and prevents chemotherapeutic oxidative phosphorylation and energy generation. Fisicaro et al. [116] reported that mitochondrion-targeted antioxidants induced a restoration of mitochondrial and antiviral CD8^+^ activities. IL-12 and IL-15 have been shown to restore the metabolic capacity of exhausted T cells by reducing their reliance on glycolysis and enhancing mitochondrial regulation, thereby improving T-cell function through oxidative phosphorylation. Quantitative analysis reveals that IL-12 and IL-15 treatment restores OXPHOS activity by approximately two-fold in exhausted HBV-specific CD8^+^ T cells, significantly improving their metabolic fitness and antiviral function [117]. Therefore, within the immunosuppressive milieu of HCC, restoring effective antitumor T-cell function may be achieved by targeting mitochondrial regulation of metabolic reprogramming in immune cells. Mitochondria have been indicated as potential therapeutic targets in chronic HBV infection, as improving MQC in virus-specific CD8^+^ T cells help support mitochondrial integrity and perform a role in preventing functional exhaustion. However, further studies are needed to establish whether this approach can effectively prevent exhaustion in humans.

Hepatocarcinogenesis is favored by persistent inflammation induced by overactivated CD8^+^ T cells, even if HBV-specific CD8^+^ T cells have a role in the antitumor immune response [118]. Hao et al. [119] reported that HBsAg-driven inflammation and hepatocarcinogenesis are strongly influenced by inappropriate CD8^+^ T-cell-mediated immune responses. Therefore, preserving immune homeostasis and tailoring therapeutic strategies to the distinct stages of HCC progression are critical for the effective treatment of HBV-related HCC.

### 6.2. MASLD-Induced HCC

MASLD eventually progresses to cirrhosis and HCC and is thought to represent a metabolic predisposition to HCC. Triglyceride buildup in the fatty liver and its progression to metabolic dysfunction-associated steatohepatitis (MASH) are characteristic histopathological expressions [120]. The buildup of inflammatory cells in the liver is often greater in MASH than in steatosis, indicating that the immune system activation contributes to fatty liver and HCC development [121]. In patients with MASH-HCC, activated yet exhausted CD8^+^ PD-1^+^ T cells have been identified. PD-1 expression in this context is generally considered a marker of T-cell exhaustion resulting from chronic antigen stimulation, though it also reflects a compensatory mechanism to limit immunopathology in the inflamed MASH liver. Regardless, the presence of PD-1 on these cells indicates a dysfunctional state, as attempts to enhance PD-1-mediated activation of intratumoral CD8^+^ PD-1^+^ T cells failed to induce tumor regression, suggesting compromised immune surveillance, likely due to aberrant T-cell activation associated with MASH [122].

A selective increase in Treg subpopulations in MASH has been observed, although with a decrease in the total number of CD4^+^ T cells. Herein, Treg suppresses the immune system and supports the growth of tumors inside the TME. Treg and Th22 cells have regulatory functions in MASH-associated HCC [123]. Wang et al. [124] found that the liver damaged by MASH showed high concentration of NETs. They subsequently confirmed that NETs interact with Tregs in the development of MASH-HCC and that NETs encourage Treg variation by promoting mitochondrial respiration. Targeting the metabolic pathway essential for Treg cells by modulating mitochondrial biogenesis during early hyper-immune activation strengthen their immunosuppressive function. Of note, treatment with DNase I, which degrades NETs, has been shown to disrupt NET-induced mitochondrial respiration in CD4^+^ T cells and reduce Treg differentiation, thereby improving immunosuppression in MASH-HCC models.

Meanwhile, MASLD is largely triggered by the disruption of lipid metabolism and the deposits of fat in the liver. Thus, limiting lipid overload in immune cells is crucial for optimal immunological function, especially in anticancer surveillance [125]. Ma et al. [126] found that when CD4^+^ T cells in the liver take in excess fat released from liver cells, this fat buildup inside the T cells triggers a specific form of cell death driven by mitochondria. Excess linoleic acid impairs hepatocyte mitochondrial activity and increases oxidative damage. Fu et al. [127] reported a novel therapeutic approach based on isolation of mitochondria from HCC cells and injection into mice with fatty liver disease induced by a high-fat diet. This procedure successfully replaced the animals’ damaged mitochondria, effectively restoring normal liver lipid metabolism. However, translating this therapy to humans presents significant challenges, including how to deliver mitochondria safely and effectively to the liver, potential immune rejection of donor mitochondria, and ensuring long-term safety. Altogether, these findings indicate that focusing on mitochondrial transfer to control CD4^+^ T cell lipid absorption is a potential approach for treating MASLD. Additional investigation is required to determine in which way to target immune cell activity at various phases of the HCC progression and whether mitochondrial transfer can be developed into a safe and feasible treatment for humans.

## 7. Immune Checkpoint Blockade and Mitochondrial Metabolic Reprogramming

**Figure 4:**
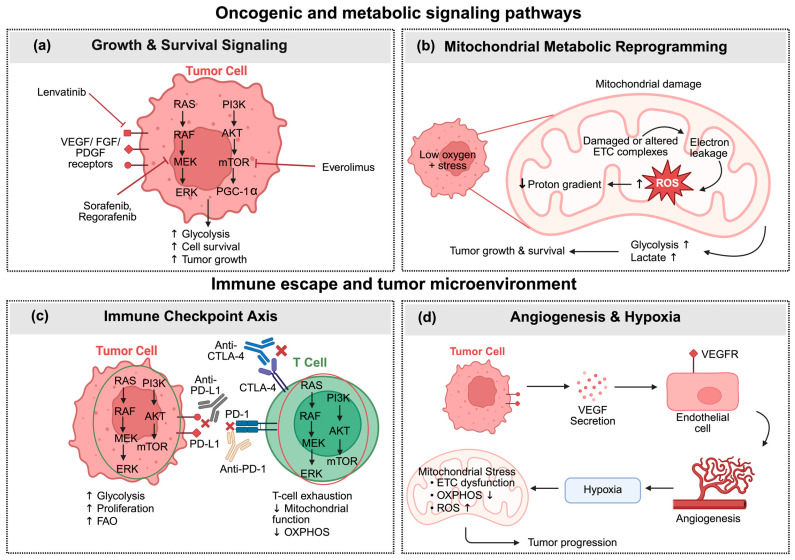
Signaling pathways involved in HCC progression, highlighting tumor cell metabolism, mitochondrial dysfunction, immune escape, and angiogenesis. (**a**) Growth and survival signaling in HCC cells. The activation of VEGFR/FGFR/PDGFR–PI3K–AKT–mTOR pathway promotes glycolysis, cell survival, and tumor growth. (**b**) Mitochondrial metabolic reprogramming in HCC cells. Mitochondrial dysfunction reduces OXPHOS, increases ROS production, and transfers energy generation toward glycolysis. (**c**) Immune escape mechanism in the tumor microenvironment. PD-L1 expressed by HCC cells binds to PD-1 on CD8^+^ T cells, leading to T-cell exhaustion and reduced mitochondrial function. (**d**) Angiogenesis-related hypoxia in HCC. VEGF-initiated blood vessel formation induces hypoxia, mitochondrial stress, and increased ROS levels. Abbreviations: AKT, protein kinase B; CTLA-4, cytotoxic T-lymphocyte associated protein-4; ERK, extracellular signal-regulated kinase; ETC, electron transport chain; FAO, fatty acid oxidation; FGF, fibroblast growth factor; PDGF, platelet-derived growth factor; HCC, hepatocellular carcinoma; MEK, mitogen-activated protein kinase kinase; mTOR, mechanistic target of rapamycin; OXPHOS, oxidative phosphorylation; PD-1, programmed death-1; PD-L1, programmed death ligand-1; PGC-1 α, peroxisome proliferator-activated receptor gamma coactivator-1 α; PI3K, phosphoinositide 3-kinase; RAF, rapidly accelerated fibrosarcoma kinase; RAS, rat sarcoma proto-oncogene; ROS, reactive oxygen species; VEGF, vascular endothelial growth factor; VEGFR, vascular endothelial growth factor receptor. Created with Biorender.com.

Immune checkpoint inhibitors (ICIs), such as anti-PD-1/PD-L1 and anti-CTLA-4 (antibody against cytotoxic T-lymphocyte-associated protein 4), contribute to controlling the expression of immune checkpoint molecules that HCC cells use to control immune system [2] (Figure 4c). Previous research showed that inhibiting PD-1 or CTLA-4 signaling increases mitochondrial activity, which is essential for T-cell function and thereby prevents T-cell exhaustion [128]. PD-1 influences T cells by upregulating the expression of CPT1A, which increases FAO of endogenous lipids and drives metabolic reprogramming from glycolysis to enhanced FAO [129]. Conversely, CTLA-4 inhibits glycolysis in T cells without increasing FAO. PD-1-engaged T cells remain metabolically active through FAO-derived energy, whereas CTLA-4 signaling suppresses T-cell differentiation, as naïve T cells require glycolysis to differentiate into effector T cells. These different effects of CTLA-4 and PD-1 on T cell metabolic reprogramming explain why their blockage has different effects on reenergizing exhausted T cells [130]. Studies have shown that factors such as the T-cell differentiation state [131], the tumor glycolytic capacity, and the local metabolic environment [132] influence the outcome of checkpoint blockade. For example, CTLA-4 blockade is more effective in glycolysis-low tumors, where it promotes metabolic control and immune cell infiltration [132]. Additionally, PD-1 blockade has been confirmed to preferentially expand TCF1^+^ progenitor exhausted CD8^+^ T cells, which rely on distinct metabolic programs [131]. Therefore, while PD-1 and CTLA-4 clearly use different metabolic pathways, the results are variable and depend on the specific context.

Immune checkpoints can simultaneously reduce tumor-cell metabolism and restore T-cell function [133]. PD-L1 signaling has been shown to directly enhance glycolysis in tumor cells by triggering the protein kinase B (AKT)/mechanistic target of rapamycin (mTOR) pathway, resulting in increased lactate production and glucose absorption, as well as the growth and survival of these tumor cells. On the other hand, a therapeutic PD-L1 inhibitor has been shown to reduce the rate of glycolysis by preventing PD-L1 from interacting with the PD-1 receptor, which restored the amount of glucose in tumor cells and slowed the growth of the tumor [134]. Other ICIs, including CD47, can prevent cancer cells from being engulfed by binding to the signal regulatory protein α (SIRPα) receptor, which is expressed on DCs and macrophages [135]. Using anti-CD47 antibodies to block the CD47-SIRPα interaction stimulated innate immunity by supporting macrophages to kill cancer cells [136]. Additionally, anti-CD47 stimulate adaptive immunity by allowing absorbed antigens to be cross-presented, mainly by DCs, resulting in antitumor cytotoxic effects. Combinatorial treatments with PD-1 antibodies can enhance this effect [137]. Beyond T cells, ICIs modulate multiple immune populations within the TME. CTLA-4 blockade promotes early T-cell priming in lymphoid tissues by enhancing CD28 co-stimulation, leading to increased CD4^+^ helper T cells and CD11c^+^ DCs. PD-1 blockade directly enhances NK cell cytotoxicity, as PD-1 expression on tumor-infiltrating CD56dim NK cells correlates with poor prognosis, and its inhibition restores NK-mediated killing [86]. A summary of preclinical studies investigating combination strategies that target metabolic pathways to enhance ICI efficacy in HCC is reported in Table 3.

The combination of anti-PD-L1 and anti-VEGF therapies significantly reduces FOXP3^+^ regulatory T cells and F4/80^+^ tumor-associated macrophages, while simultaneously increasing CD8^+^ cytotoxic T cells and Granzyme B-positive cells. MDSCs, which inhibit T-cell activation and promote immunosuppression through arginase-1 and ROS production, are also reduced by effective ICI drugs [86].

Nivolumab blocks the PD-1 receptor on T-cells. This blockade responds to the PD-1-induced metabolic reprogramming that forces T-cells into a state dependent on FAO [144]. By inhibiting PD-1, nivolumab restores glycolytic capacity and mitochondrial function in T-cells, reversing exhaustion and enhancing their anti-tumor activity [134]. It also indirectly affects tumor cell metabolism by disrupting the PD-L1/AKT/mTOR/glycolysis axis. A phase I/II trial established the efficacy of nivolumab in patients with advanced HCC, including those previously treated with sorafenib [145]. Like nivolumab, pembrolizumab is an anti-PD-1 antibody that strengthens T-cell function by improving metabolic limitations (reduced glycolysis, enforced FAO) imposed by PD-1 signaling. This restoration of mitochondrial bioenergetics in T-cells is fundamental to its clinical effect [146]. A phase II trial evaluating pembrolizumab in patients with advanced HCC who had previously been treated with sorafenib. Pembrolizumab (anti-PD-1) (200 mg every 3 weeks) has shown clinically meaningful antitumor activity, supporting the role of PD-1 blockade as a therapeutic option in pretreated HCC patients [147]. Atezolizumab (anti-PD-L1) blocks PD-L1 on tumor cells. Directly, this prevents PD-1 engagement on T-cells. It also disrupts the PD-L1-driven AKT/mTOR pathway in HCC cells themselves, thereby inhibiting the tumor’s glycolytic metabolism and lactate production [148]. Bevacizumab (anti-VEGF) complements this by normalizing the tumor vasculature and potentially improving T-cell infiltration [149]. Results from phase 3 trial showed that atezolizumab plus bevacizumab improved the overall survival and progression-free survival compared with sorafenib in unresectable HCC. The combination reduced the risk of death by 42% and demonstrated a higher 12-month survival rate [150].

ICIs have shown encouraging results about antitumor effects, but many HCC patients do not respond to these drugs. This is likely due to several mechanisms that inhibit the efficiency of antitumor immunity under tumor metabolic and microenvironmental conditions. For these patients, new combinatorial approaches are required [90]. Chamoto et al. [71] showed that physiological levels of ROS were able to act as signaling molecules and strongly enhance mitochondrial function in tumor-reactive T cells. When combined with PD-1 blockade, this produced synergistic antitumor effects. This was achieved by activating mTOR and AMP-activated protein kinase (AMPK) phosphorylation and upregulating PGC-1α, a downstream target of both pathways that promotes mitochondrial activity. They also discovered that the anticancer effects of PD-1 blocking treatment were synergistically improved by activators of AMPK, mTOR, or PGC-1α. For patients whose condition is not sensitive to PD-1 inhibitors, these results offer a proof for a combined approach incorporating mitochondrial activation drugs and PD-1 inhibitors. These results also show that indicators of mitochondrial activation, such as PGC-1α, may help determine how well PD-1 blockage works as an anticancer treatment. Another study [151] suggested a direct relationship between PGC-1α and PD-1, showing that PD-1 inhibits the activity and expression of PGC-1. This could help clarify why reintroducing PGC-1α overcomes T-cell depletion. Tan et al. [152] showed that the antibiotic tigecycline holds potential as a repurposed drug for HCC by selectively targeting HCC cells and inhibiting mitochondrial protein translation. This disrupts organelle’s function and triggers lethal oxidative damage. Importantly, this mitochondrial targeting strategy has been shown to enhance the effectiveness of the chemotherapy drug cisplatin against HCC in preclinical models.

The gut microbiota also plays a role in HCC progression [153]. Beneficial bacteria such as *Bifidobacterium* have been shown to enhance the efficacy of chemotherapy and immunotherapy by improving mitochondrial metabolism in T cells and promoting antitumor immunity [154]. In contrast, gut dysbiosis, characterized by an imbalance in microbial composition, promotes inflammation, compromises intestinal barrier integrity, and accelerates tumor progression [155]. Microbiota-derived metabolites, including short-chain fatty acids and bile acids, influence mitochondrial function in both cancer cells and immune cells, positioning the gut–liver axis as a potential therapeutic target [156].

Similarly, TILs encourage FAO as a means of preservation in hypoxic and hypoglycemia TME, where immune cells and tumor cells compete for nutrition. Consequently, CD8^+^ TILs maintain their energy and effector activities. On the other hand, PD-1 inhibitors do not directly affect TIL function by promoting FAO; instead, they reduce tumor development by reactivating exhausted TILs that have been suppressed. While this reactivation involves metabolic reprogramming, it is important to distinguish between the tumor’s and the therapy’s effect. Increased FAO and PD-1 inhibitors improve immunotherapy’s efficacy and slow the development of tumors [157]. In conclusion, PD-1 inhibitors have a significant impact on mitochondria. Like memory T cells maintaining survival by FAO, PD-1 inhibition in CD8^+^ T cells causes metabolic reprogramming of T cells that depends on FAO, which could be responsible for the long survival of PD-1-stimulated T cells. Additionally, PD-1 signaling has a significant impact on the shape and function of the mitochondria. Immune checkpoint molecules induce a variety of structural alterations in mitochondria, such as decreases in both the length and quantity of mitochondrial cristae after PD-1 stimulation [158]. This indicates how crucial it is for memory T cells to maintain their mitochondrial structure and function.

Collectively, immune checkpoint signaling interconnects with oncogenic and metabolic pathways to shape both tumor progression and antitumor immunity in HCC. Activation of VEGFR/FGFR/PDGFR (vascular endothelial growth factor receptor/fibroblast growth factor receptor/platelet-derived growth factor receptor)-initiated PI3K-AKT-mTOR (phosphoinositide 3-kinase–protein kinase B–mechanistic target of rapamycin) and RAS-RAF-MEK-ERK (Rat sarcoma-rapidly accelerated fibrosarcoma-mitogen-activated protein kinase kinase–extracellular signal-regulated kinase) cascades promotes metabolic reprogramming in tumor cells, enhancing glycolysis, cell survival, and tumor growth [90] (Figure 4a). Similarly, mitochondrial metabolic alterations, marked by electron transport chain dysfunction, increased ROS production, and disrupted proton gradients under hypoxic stress, shift HCC cells toward glycolytic dependence, further supporting tumor progression (Figure 4b). In parallel, tumor-derived VEGF stimulates angiogenesis via endothelial VEGFR signaling, while hypoxia induces mitochondrial stress, impaired oxidative phosphorylation, and elevated ROS within the TME, collectively fostering immune suppression and disease advancement (Figure 4d). These connected processes highlight how mitochondrial dysfunction and metabolic remodeling not only sustain tumor growth but also limit immune cell efficacy, highlighting the therapeutic potential of combining immune checkpoint blockade with metabolic or mitochondrial-targeted strategies.

## 8. Conclusions

HCC remains a fatal cancer with limited therapeutic options, especially in advanced stages. A deeper investigation of its biology indicates that the TME and its resistance to treatment may also be linked to fundamental cellular processes initiated by mitochondria. In HCC, dysfunctional MQC, including imbalanced dynamics, reduced biogenesis, and impaired degradation, helps cancer cells to survive and grow. This dysfunction promotes metabolic reprogramming towards glycolysis, generates damaging ROS, and allows cells to evade programmed death, directly increasing tumor growth and resilience. At the same time, the nutrient-poor TME disrupts mitochondrial function in immune cells, damaging mitochondria in anti-tumor T cells and NK cells while supporting pro-tumor Tregs and MDSCs. Therapeutic strategies prioritizing cell-specific metabolic interventions such as PGC-1α activators (e.g., AMPK agonists, 4-1BB agonists) to enhance mitochondrial function in exhausted CD8^+^ T cells and NK cells, paired with FAO blockers (e.g., etomoxir, FABP5 inhibitors) to selectively disable immunosuppressive Tregs and MDSCs, should be explored. Such rational combinations and their integration with ICIs may hold promise for improving outcomes in HCC patients.

## Figures and Tables

**Table 1:** Comparative expression of mitochondrial biogenesis and genomic alterations in HCC versus normal liver.

Marker	Expression in Normal Liver	Expression in HCC	Functional Implication	Ref.
PGC-1α	High	↓ 30–50%	Reduced OXPHOS capacity; metabolic reprogramming toward glycolysis	[27]
TFAM	Baseline	↓ 25–30%	Reduced mtDNA copy number; impaired mtDNA transcription and maintenance	[28]
NRF1	Moderate	↓ 20–40%	Reduced nuclear-mitochondrial communication; reduced expression of OXPHOS subunits	[29]
TERT promoter	Wild-type	44% mutation frequency	Telomerase reactivation; enhanced cell survival and proliferation	TCGA
TP53	Wild-type	Frequent mutations; also inhibited by MDM4 overexpression	Loss of tumor suppressor function; impaired DNA damage response and apoptosis	TCGA
CTNNB1	Wild-type	Frequent mutations	Constitutive Wnt pathway activation; promotion of cell growth and differentiation	TCGA
Immune evasion genes	Baseline	20% show elevated expression	Prediction of sensitivity to immunotherapies	TCGA

Abbreviations: CTNNB1, catenin beta 1; MDM4, murine double minute 4; mtDNA, mitochondrial DNA; NRF1, nuclear respiratory factor 1; OXPHOS, oxidative phosphorylation; PGC-1α, peroxisome proliferator-activated receptor gamma coactivator 1-alpha; TCGA, The Cancer Genome Atlas; TERT, telomerase reverse transcriptase; TFAM, mitochondrial transcription factor A; TP53, tumor protein p53; Wnt, Wingless/Int-1.

**Table 2:** Metabolic profiles of key immune cells in the healthy state, their dysfunction within the HCC TME, and potential strategies to restore their antitumor activity.

Cell Type	Healthy Metabolism	TME Dysfunction	Therapeutic Strategy
CD8^+^ T cells	Glycolysis + OXPHOS for effector function; FAO for memory [53]	PGC-1α downregulation, mitochondrial depolarization, exhaustion [54]	4-1BB agonists (induce fusion/biogenesis independent of PGC-1α); PGC-1α overexpression [55]
NK cells	OXPHOS dominant at rest; both glycolysis and OXPHOS upon activation [56]	TGF-β-induced mitochondrial fragmentation; reduced glycolysis (↓ 60% in HCC)	Mitochondrial transplantation [57]; TGF-β blockade; mito-targeted IL-15
M1 Macrophages	Glycolysis with fragmented mitochondria [58]	M2 polarization under low glucose, high lactate [58]	CSF-1R blockers to reduce TAM recruitment; metabolic reprogramming toward glycolysis

Abbreviations: CD8^+^ T cell, cluster of differentiation 8 positive T cell; FAO, fatty acid oxidation; HCC, hepatocellular carcinoma; IL-15, interleukin-15; M1, classically activated macrophages; NK cell, natural killer cell; OXPHOS, oxidative phosphorylation; PGC-1α, peroxisome proliferator-activated receptor gamma coactivator 1-alpha; TAM, tumor-associated macrophage; TGF-β, transforming growth factor beta; TME, tumor microenvironment.

**Table 3:** Preclinical studies targeting metabolic pathways to enhance ICI efficacy in HCC.

Target/Mechanism	Combined Treatment	Experimental Model	Key Findings	Reference
MCT4 (lactate transporter)	VB124 + anti-PD-1	Murine HCC models	MCT4 inhibition synergizes with PD-1 blockade to improve therapeutic efficacy and extend survival in HCC-bearing mice.	[138]
MCT (lactate transporter)	MCT inhibitor + anti-PD-1	Murine HCC models	Blocking lactate transport enhances the anti-tumor effects of PD-1 immunotherapy in hepatocellular carcinoma.	[139]
GLUT1 (glucose transporter)	Citalopram + anti-PD-1	Murine HCC models	Citalopram selectively targets highly glycolytic liver tumors and potentiates the response to anti-PD-1 treatment.	[140]
HIF-1/GLUT1 axis	GJB2 knockdown + anti-PD-1	Murine HCC models	Silencing GJB2 suppresses HIF-1-mediated GLUT1 expression and increases sensitivity to PD-1 blockade.	[141]
SRSF10/MYB/glycolysis pathway	1C8 (SRSF10 inhibitor) + anti-PD-1	Murine HCC models	Pharmacological inhibition of SRSF10 with 1C8 suppresses glycolysis, delays tumor progression, and enhances anti-PD-1 response.	[142]
CircRHBDD1/YTHDF1/PIK3R1 axis	CircRHBDD1 inhibition + anti-PD-1	Murine HCC models	Targeting circular RNA CircRHBDD1 reduces glycolytic activity and synergizes with PD-1 blockade to suppress tumor growth and improve survival.	[143]

Abbreviations: CircRHBDD1, circular RNA rhomboid domain containing 1; GJB2, gap junction protein beta 2; GLUT1, glucose transporter 1; HCC, hepatocellular carcinoma; HIF-1, hypoxia-inducible factor 1; MCT, monocarboxylate transporter; MYB, MYB proto-oncogene transcription factor; PD-1, programmed cell death protein 1; PIK3R1, phosphoinositide-3-kinase regulatory subunit 1; SRSF10, serine and arginine rich splicing factor 10; YTHDF1, YTH N6-methyladenosine RNA binding protein 1.

## Data Availability

No new data were created or analyzed in this study. Data sharing is not applicable to this article.
